# Molecular subtyping bladder cancer: Is it ready for clinical practice?

**DOI:** 10.3906/sag-2004-200

**Published:** 2020-11-03

**Authors:** Funda VAKAR LOPEZ

**Affiliations:** 1 Department of Pathology, HMC Anatomic Pathology, University of Washington, Seattle USA

**Keywords:** Bladder cancer, molecular subtypes, urothelial carcinoma

## Abstract

Bladder cancer, one of the more common cancers, is a heterogeneous disease, both morphologically and clinically. Although histological classification and extent of the disease (staging) guide treatment options, the heterogeneity in responses to therapy highlights the need for better stratification of patients for the appropriate therapy in order to achieve better outcomes. Several molecular classifications of muscle-invasive bladder cancer have been proposed but currently their use in clinical practice is limited by the complexity of the methods used and diversity of the subtypes.

Bladder cancer is among the most prevalent and deadly cancers with approximately 550K new cases and 200K deaths in 2018 worldwide. Although there have been some advances in treatments over the years, there is still a need for a more refined and tailored (targeted) therapy options.

The vast majority of bladder cancers are of urothelial origin. The classification of urothelial tumours in the recent 2016 World Health Organization (WHO) Classification of Tumours of the Urinary System and Male Genital Organs in part parallels the dual track concept of carcinogenesis [1]. Most urothelial carcinomas have a papillary architecture and do not invade the bladder wall. Classified as noninvasive papillary urothelial carcinomas, they are treated by local excision and bladder preserving intravesical therapies. Although some of these tumours progress as invasive tumours, most recur as noninvasive tumours. Since standard long-term management entails periodic cystoscopies with biopsies, as needed, bladder cancer is one of the most expensive cancers to treat. A major challenge is to find effective treatment strategies for high grade, high stage invasive tumours for which the current 5-year survival rate ranges between 36%, when there are lymph node metastasis, and 5% for distant metastasis.

Urothelial carcinomas are histomorphologically dissimilar. Due to the inherent plasticity of the urothelium, urothelial carcinomas include many histologic variants, such as micropapillary, plasmacytoid/diffuse/signet ring, lipoid, etc., as well as different states of histologic differentiations, i.e. squamous and glandular. The variant morphologies guide some therapeutic decisions. However, for many tumours precise targetable markers predictive of response to a specific therapy have not been identified. In recent years molecular characterizations of urothelial carcinoma [2–4] have defined subtypes of muscle invasive cancer that respond differently to current chemotherapy regimens [5]. These studies showed that muscle invasive bladder cancer (MIBC) has a high mutation rate similar to that of melanoma and nonsmall cell lung cancers. One limitation is that the data have been derived from largely nonoverlapping data sets, using different methods and resulting in multiple nomenclatures for the subtypes. In order to achieve an international consensus on the MIBC molecular subtypes, the latest study [6] analysed the published classification schemes and further stratified previously defined categories of luminal and basal (similar to the breast carcinoma) into 6 distinct subtypes: luminalpapillary (LumP), luminal nonspecified (LumNS), luminal unstable (LumU), stroma-rich, basal/squamous (Ba/Sq), and neuroendocrine-like (NE-like).

Each subtype has distinctive molecular features, progression-risk and responsiveness to specific systemic drugs.

· LumP subtype (24%) is enriched in the noninvasive (pTa) phenotype (papillary architecture with low risk for progression) and is strongly associated with FGFR3 transcriptional activity. These tumours may respond to neoadjuvant cisplatin-based chemotherapy as well as to pan-FGFR inhibitor agents, i.e. infigratinib, irrespective of the mutation or translocation status of FGFR3. 

 · The LumNS (formerly known as “luminal infiltrated”) subtype (8%) has an elevated stromal infiltration signature, mainly myofibroblastic. It also expresses CD274 (PD-L1) and CTLA4 markers, defining a phenotype that is reported to respond to immune checkpoint therapy (atezolizumab).

· The LumU subtype (15%) is a recently defined category characterized by expression of luminal markers (uroplakins) as well as by KRT20 and SNX31. Being a new category, no therapy response information has been acquired or reported.

· Stroma-rich subtype (15%) has intermediate levels of urothelial differentiation. This subtype is also characterized by stromal infiltration with overexpression of smooth muscle, endothelial, fibroblast, and myofibroblast gene signatures as well as overexpression of mainly B- and T-cell markers.

· The Bas/Sq subtype (35%), more often seen in women than in men, exhibits squamous differentiation and expresses basal keratin(s) and immune markers (cytotoxic lymphocytes and natural killer cells). Both cisplatin-based neoadjuvant chemotherapy (NAC) and immune therapy are appropriate treatment options for this subtype.

· The sixth subtype NE-like (3%) expresses neuroendocrine and neuronal genes and has a high proliferative state. Concurrent TP53 and RB1 inactivation is common in NE-like tumours. As in neuroendocrine neoplasms arising in other sites, these tumours may benefit from etopside-cisplastin therapy.

Although subtype-specific personalized therapies could optimize patient outcomes, the subtype-drug correlation needs to be verified in prospective studies before they can be integrated into clinical practice. Hurdles to widespread implementation of subtyping bladder carcinoma include the difficulty of obtaining high quality RNA and the labour and cost of microarray analyses with expertise in bioinformatics.

A possible solution to these hurdles is identifying subtype-specific markers that can be more easily and readily detected by immunohistochemistry (IHC) using analyte-specific primary antibodies which reflect the molecular subtypes.

Several studies [7,8] compared the performance of antibody panels (composed of 5 to 28 antibodies) and with the transcriptome profiles of the same cohort of analytes. The results showed that GATA3 and CK5/6 identified luminal and basal subtypes, respectively, with 91% accuracy based on transcriptome analysis. Using a panel of 28 antibodies, the Lund group [8] showed that some tumours of different IHC-defined groups clustered together; conversely, some of the IHC-defined groups clustered separately. These discrepancies may be due to contamination of samples by nontumour cells in the transcriptome and/or interpatient and intra-patient tumour heterogeneity. Currently, there is not enough evidence to molecularly or phenotypically subtype bladder cancers. However, the proposed molecular and/or immunohistochemical subtyping could be used for building a framework for prospective hypothesis generation and hypothesis testing in clinical trials.
